# Distribution of airway narrowing responses across generations and at branching points, assessed *in vitro *by anatomical optical coherence tomography

**DOI:** 10.1186/1465-9921-11-9

**Published:** 2010-01-22

**Authors:** Peter B Noble, Robert A McLaughlin, Adrian R West, Sven Becker, Julian J Armstrong, Peter K McFawn, Peter R Eastwood, David R Hillman, David D Sampson, Howard W Mitchell

**Affiliations:** 1 Division of Clinical Sciences, Telethon Institute for Child Health Research, (Roberts Road), Perth, (6008), Australia; 2 Physiology, School of Biomedical, Biomolecular and Chemical Sciences, University of Western Australia, (Stirling Highway), Perth, (6009), Australia; 3 Optical + Biomedical Engineering Laboratory, School of Electrical, Electronic and Computer Engineering, University of Western Australia, (Stirling Highway), Perth, (6009), Australia; 4 School of Anatomy and Human Biology, University of Western Australia, (Stirling Highway), Perth, (6009), Australia; 5 Department of Pulmonary Physiology, Sir Charles Gairdner Hospital, (Hospital Avenue), Perth, (6009), Australia; 6 West Australian Sleep Disorders Research Institute, Sir Charles Gairdner Hospital, (Hospital Avenue), Perth, (6009), Australia

## Abstract

**Background:**

Previous histological and imaging studies have shown the presence of variability in the degree of bronchoconstriction of airways sampled at different locations in the lung (i.e., heterogeneity). Heterogeneity can occur at different airway generations and at branching points in the bronchial tree. Whilst heterogeneity has been detected by previous experimental approaches, its spatial relationship either within or between airways is unknown.

**Methods:**

In this study, distribution of airway narrowing responses across a portion of the porcine bronchial tree was determined *in vitro*. The portion comprised contiguous airways spanning bronchial generations (#3-11), including the associated side branches. We used a recent optical imaging technique, anatomical optical coherence tomography, to image the bronchial tree in three dimensions. Bronchoconstriction was produced by carbachol administered to either the adventitial or luminal surface of the airway. Luminal cross sectional area was measured before and at different time points after constriction to carbachol and airway narrowing calculated from the percent decrease in luminal cross sectional area.

**Results:**

When administered to the adventitial surface, the degree of airway narrowing was progressively increased from proximal to distal generations (r = 0.80 to 0.98, P < 0.05 to 0.001). This 'serial heterogeneity' was also apparent when carbachol was administered via the lumen, though it was less pronounced. In contrast, airway narrowing was not different at side branches, and was uniform both in the parent and daughter airways.

**Conclusions:**

Our findings demonstrate that the bronchial tree expresses intrinsic serial heterogeneity, such that narrowing increases from proximal to distal airways, a relationship that is influenced by the route of drug administration but not by structural variations accompanying branching sites.

## Introduction

Airways are structurally complex and *in vivo *are subject to mechanical and physiological factors which potentially lead to differences in the degree of narrowing in response to a comparable provocative stimulus, often referred to as airway 'heterogeneity'. Structural and functional heterogeneity, which impacts on normal respiratory function, could be present across different generations of the bronchial tree, and notably at branching points where the parent airway gives rise to daughter generations. There is a need to better characterize airway heterogeneity as it is thought to be important in the pathophysiology of obstructive pulmonary disease [1,2].

The method used to measure airway narrowing is critical to the identification of heterogeneity. Several studies based on direct imaging have compared the extent of airway narrowing across different airways in the lung [3-8], and nearly all reported some heterogeneity in response to bronchoconstrictor challenge. Other studies have used geometrical analyses of airway smooth muscle (ASM) length in fixed airway tissue to assess pre-mortem airway narrowing, and also report considerable heterogeneity amongst the airways sampled [9-12]. In contrast, the degree of heterogeneity in airway responses is not apparent from global measures of lung function such as forced expiratory volume in 1 sec (i.e., FEV_1_) or airway resistance, leaving the contribution from different airway generations unknown.

Whilst heterogeneity in airway narrowing responses has been frequently observed, it remains unclear whether the amount and rate of airway narrowing are randomly distributed throughout the lung, or whether the response varies systematically along axial pathways, such as may occur with a serially distributed heterogeneity. *In vivo*, narrowing increases from the trachea to the major bronchi [8,13], presumably reflecting the transition from partial to more complete encirclement of ASM in the airway wall. Imaging of isolated airway or lung preparations either shows greatest narrowing in the smallest diameter airways [7] or in mid-sized airways [14] while histological studies identify large airways as the site of greatest narrowing [10,11]. Some of the inconsistencies and uncertainties in reports of heterogeneity are likely related to methodological complexities and limitations. In some studies for instance, aerosol deposition of bronchoconstrictor agonists could produce variable levels of ASM activation between airways [11,12] thereby influencing the relationship between airway narrowing and generation. Moreover, previous imaging and histological approaches provide information on bronchoconstriction only in a single cross sectional plane of selected airways, rather than on the three-dimensional (3D) response of contiguous parts of the bronchial tree needed to provide a comprehensive and reliable assessment of airway narrowing.

In contrast to heterogeneity between different airway generations, the potential for more localised physiological variability at the branching points of airways appears to have been overlooked. The structural design at the bifurcation deviates from the rest of the airway reflected by differences in the shape and size of cartilage plates as well as the orientation of ASM fibres [15-18]. Despite this structural complexity at branching regions it is unknown whether airway narrowing differs compared to the main body of an airway.

This study determined the distribution of airway narrowing along a part of the bronchial tree comprising a series of contiguous conducting airways spanning a range of airway generations, including associated side branches. To measure changes in airway calibre we used a recent optical imaging technique, anatomical optical coherence tomography (*a*OCT), that incorporates a moving (rotating and translating) probe and so enables the airway to be viewed across a predetermined distance such that the organ is imaged essentially in three dimensions. Unlike all previous studies, *a*OCT enabled dynamic narrowing responses of different generations of airway and side branches, in the same preparation, to be recorded contemporaneously and under identical experimental conditions. Specifically we recorded airway narrowing to carbachol, in a portion of the bronchial tree that was separated from the lung parenchyma so that any differences could be attributed to the properties of the airway alone and not to surrounding lung tissue. Carbachol was chosen as the agonist since regional differences in narrowing or the kinetics of response are independent of enzymatic breakdown. By adding carbachol directly to the fluid bathing the adventitia or lumen of the airways, we were also able to control the route of drug delivery and the doses to which the ASM was exposed.

## Methods

### Animal Handling

All animal experiments were approved by institutional ethics and animal care unit. Eight White Landrace pigs (30 ± 2 Kg), were sedated with tiletamine/zolazepam (4.4 mg/Kg im.) and xylazine (2.2 mg/Kg im.) and exsanguinated under pentobarbitone sodium anesthesia (30 mg/Kg iv.). Lungs were then removed and transported on ice to the laboratory for dissection of airways.

### Airway Preparation

**Figure 1:**
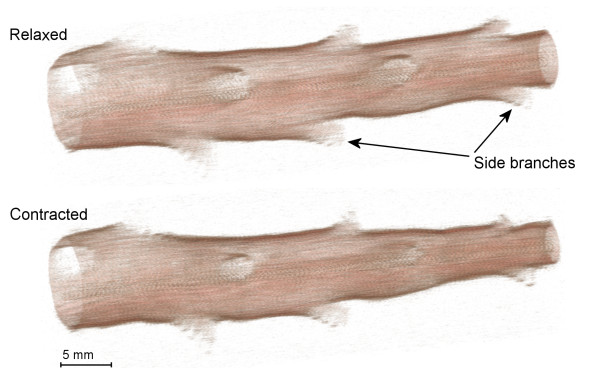
**A 3D profile of a porcine bronchial tree acquired by anatomical optical coherence tomography (*a*OCT)**. The major portion of the bronchial tree comprises a large parent bronchus that gives rise to smaller daughter bronchi (i.e., side branches) that branch off at an obtuse angle of departure. The figure shows the airway preparation in its relaxed state and after bronchoconstriction in response to carbachol.

A length of the bronchial tree was dissected from the right lower lobe of the lung, beginning from the lobar bronchus and extending distally ~5-6 cm. In the pig lung, the first 6-7 cm of bronchus comprises a large parent bronchus that runs axially with minimal bending, giving rise to daughter airways (side branches) at regular intervals (i.e., monopodial branching). The daughter airways are smaller than the parent bronchus and typically branch off with a high angle of departure. As each side branch was reached it was cleared of parenchyma over a distance of ~1 cm and ligated at a distance furthest from the parent bronchus. Airway generation was determined by counting the number of side branches arising from the parent bronchus, taking the trachea as generation #0. The dissection produced a straight, tapering and cylindrical bronchial tree spanning generations #1 to #13. A 3D rendering of an airway preparation imaged with *a*OCT is shown in Figure 1.

Airway preparations were cannulated at both ends and placed horizontally in an organ bath containing gassed (95% O_2_, 5% CO_2_) Krebs solution (mM: NaCl 121; KCL 5.4; MgSO_4 _1.2; NaHCO_3 _25; sodium morpholinopropane sulphonic acid buffer 5.0; glucose 11.5; and CaCl_2 _2.5) at 37°C. The preparation was stretched slightly to a length shown previously to approximate functional residual capacity in the pig lung, i.e., ~105% of the fully deflated length at 0 cmH_2_O [19]. Intraluminal pressure was 5 cmH_2_O, set by the height of a reservoir containing Krebs solution connected at the distal side of the preparation.

### Anatomical Optical Coherence Tomography

Airway dimensions were measured using anatomical optical coherence tomography (*a*OCT) [20-23]. During *a*OCT imaging, low coherence near infra-red light is emitted from an optical probe. The same probe is used simultaneously to detect reflections of light from the air-tissue interface of the lumen which allows the distance to the luminal surface to be determined by interferometry. By rotating the probe, a 2D axial image of the lumen may be reconstructed, and by precisely moving the probe backwards or forwards using a motorized translation stage, these axial images may be combined into a 3D data set. Airway measurements using *a*OCT are calibrated to account for the refractive index of the medium, e.g., air or liquid. Airway segments in the present study were filled with Krebs solution which we determined to have a refractive index of 1.37.

The *a*OCT system has an axial resolution (optical coherence length in air) of 32.9 μm, and a minimum beam waist of 100 μm occurring 6.6 mm from the probe head. The depth of focus of the probe was 11 mm. For the present study, the rotating *a*OCT probe was encased in a transparent catheter (OD 2.2 mm). The catheter was inserted into the airway lumen, beginning at the cannula in the proximal end of the airway and extended down to the internal edge of the distal airway cannula where it was locked in position. The airway lumen was sealed by wrapping silicon tape around the catheter at its point of insertion at the proximal side of the airway. The *a*OCT probe was rotated at ~0.8 Hz, acquiring quantitative images of lumen cross sectional area, which were displayed in real time on a computer monitor. For 3D airway assessment, the probe was slowly drawn back along the length of the airway (referred to as a 'pullback scan'), constructing a 3D model of the airway (Figure 1) under specified conditions described below.

### Experimental Protocol

Before experimentation airway preparations were allowed 1 hour to equilibrate to organ bath conditions during which the lumen and adventitia of the preparation were regularly flushed with fresh Krebs solution. Tissue viability was confirmed by airway contractions to electric field stimulation (60 V, 5-ms pulse width, 30 Hz) and acetylcholine (ACh; 10^-3^M) followed by a recovery period of at least 1 hour.

Airway narrowing was assessed across the full range of generations incorporated in the airway preparation. Two protocols were used to measure airway narrowing to the bronchoconstrictor agent carbachol. In the first protocol, carbachol was administered to the adventitial surface (outside) of the airway preparation with a final bath concentration of ~1 × 10^-6 ^to 1 × 10^-5^M sufficient to produce ~50% bronchoconstriction (decrease in luminal cross sectional area, see below Analysis and Statistics). Pullback *a*OCT scans were performed in the relaxed airway before the addition of carbachol to the bath and then at 5, 15 and 30 minutes after carbachol, which approximated the time course of bronchoconstriction (see Results). The rate of pullback was 0.19 mm/sec. In the second protocol, carbachol was applied to the lumen (inside) of the airway. To achieve a comparable level of bronchoconstriction the dose of carbachol administered to the lumen (3 × 10^-4^M) was 30-300 fold greater than that applied to the adventitial surface due to the high integrity of the epithelial barrier [24]. Furthermore, as the rate of narrowing to luminally applied drugs in pig airways is typically greater than by advential drug application [24], likely due to the relatively thin internal airway wall barrier, the pullback rate was increased to 0.68 mm/sec and scans performed at 2, 5, 8, 11 and 14 minutes after the addition of luminal carbachol. Pullback scans were initiated from the distal side of the preparation which meant a systematic delay in scanning of proximal regions relative to distal regions. Due to the different pullback rates, the proximal region of the airway was scanned ~3 min later than the most distal point during adventitial carbachol application, whereas with the luminal protocol in which scanning speeds were greater the time interval was reduced to ~1 min.

At the conclusion of the experiment, airways were fixed in the bath, frozen in Tissue Tek embedding media and cryo-sectioned for staining with a Servio Stain kit (Royal Perth Hospital).

### Analysis and Statistics

Luminal cross sectional area was measured at different locations in airways before and after the addition of carbachol, and airway narrowing was calculated from the percentage decrease in luminal cross sectional area. Unless otherwise stated, airway cross sectional area was measured by tracing an area around the lumen using custom designed quantification software developed in the C++ language.

**Figure 2:**
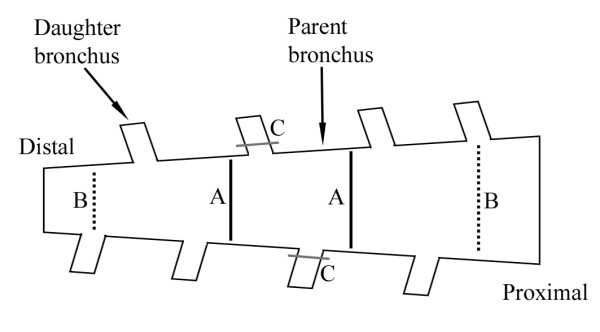
**A schematic of the airway preparation indicating the measurements performed**. Indentified in the figure are the parent bronchus and a connecting daughter bronchus (i.e., side branch). The distal and proximal ends of the airway preparation are also labeled. For the study, three separate analyses were performed: (Analysis 1), narrowing in the parent bronchus was measured and compared between generations (A, black line), away from regions of branching; (Analysis 2), narrowing in the parent bronchus was measured at the midpoint of branching, where the parent bronchus was seen to open into a daughter side branch (B, dotted black line), and it was compared to narrowing measured immediately proximal and distal to the branching point; (Analysis 3), narrowing within the 'mouth' of daughter side branches was measured (C, grey line) and compared to narrowing within the adjacent parent bronchus at the same site (B).

Three separate analyses were performed. For the first analysis (Analysis 1), we compared airway narrowing between generations, at sites located away from branching points (see schematic in Figure 2). The airway generations studied were in the range of #3-11, although due to differences in branching patterns between airways, most notably points of dual side branches in some airways, the number of measurements made in each airway was not always identical. Measurements were taken along the airway preparation, avoiding regions closer than 1 cm to the plastic cannula at either end of the airway to eliminate any restriction on narrowing by the cannula insert.

In addition to assessing differences in airway narrowing between generations, this study also determined whether there were local inhomogeneities in narrowing at regions of airway branching. Two additional analyses were carried out to assess this effect: (Analysis 2) Airway narrowing within the parent bronchus was measured at the midpoint of branching, i.e., where the parent bronchus was seen to open into a daughter side branch (Figure 2), and this was compared to narrowing measured immediately proximal and distal to the branching point. Effects of adventitial and luminally applied carbachol were examined; (Analysis 3) Airway narrowing within the 'mouth' of daughter side branches was measured (Figure 2) and compared to narrowing within the adjacent parent bronchus. In order to measure cross sectional areas in the 'mouth' of a daughter bronchus, it was necessary to take into consideration the angle of pitch at which the daughter bronchus branched from the parent. This was achieved by constructing a 3D profile of the airway preparation (Figure 1) using successive 2D images acquired by *a*OCT pullback scans. For each daughter side branch, we manually identified the optimal oblique plane (relative to the parent bronchus) in which to make the cross-sectional area measurements. This was the plane containing the mouth of the daughter side branch. To eliminate errors caused by any change of angle between the relaxed and contracted airways, this oblique plane was identified separately for each *a*OCT acquisition. Effects of luminal carbachol only were examined. VolView software (Kitware Inc., NY, USA) was used for 3D reconstruction of airways and for the subsequent measurement of luminal cross sectional area in parent and daughter bronchi.

Graphical presentation and statistical analyses of data were achieved using Graphpad Prism (v4.03, GraphPad Software, CA, USA) and Statistica (99 Edition, StatSoft Inc., OK, USA). Airway narrowing at different anatomical locations (e.g., different airway generations or at branching points) was compared using two-way ANOVA and Newman-Keuls post hoc analyses with anatomical location and time as repeat measure variables. Time to 50% narrowing was computed for both outside and inside application of carbachol and used as an index of the rate of airway narrowing. Maximal airway narrowing/time to 50% narrowing (rate) in response to outside or inside application of carbachol were compared at proximal and distal locations using two-way ANOVA and Newman-Keuls post hoc analyses, with route of drug administration as a non repeat measure variable, and anatomical location as a repeat measure variable. Linear correlations between maximum narrowing and time to 50% narrowing were computed using Pearson's correlation analysis. Comparisons between airway narrowing measured in connecting daughter-parent bronchi were made using Student's paired t-test. Data are means ± standard error where N equals the number of animals/airway preparations, or where stated, N refers to the number of data points (see Results). A P-value < 0.05 was considered statistically significant.

## Results

**Figure 3:**
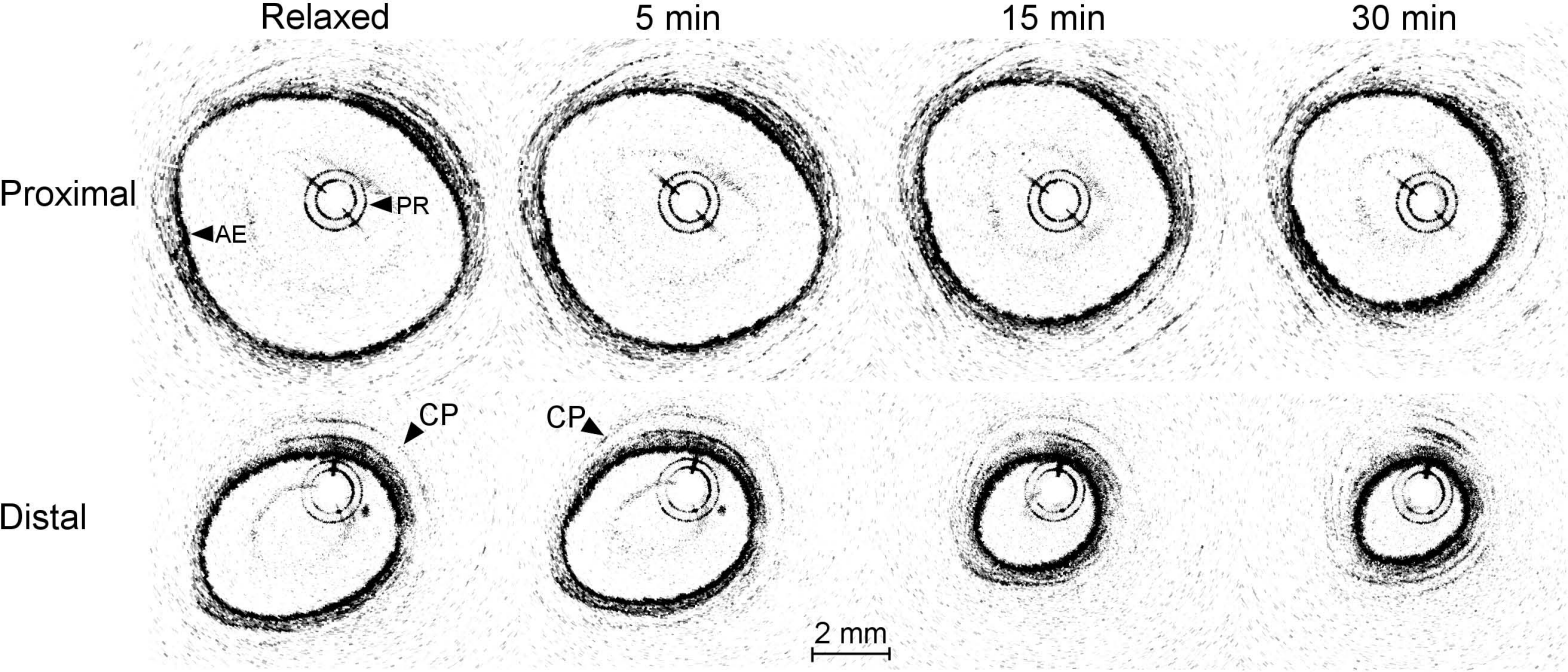
**Cross sectional images recorded by *a*OCT in a proximal and distal airway**. Proximal airway is Gen #3 and distal airway Gen #9. The airway epithelium (AE), probe catheter (PR) and cartilage plates (CP) are indentified. The airway preparation was scanned initially in its relaxed state and then 5, 15 and 30 min after addition of carbachol administered to the adventitial airway surface. Airway narrowing was typically greater and more rapid in distal airways.

Example cross sectional images of airways recorded by *a*OCT are shown in Figure 3. The figure shows 2D images of a proximal and distal airway before and after the addition of carbachol to the adventitial surface. The luminal surface of airways is indicated as well as the location of the optical probe. The relaxed lumen diameter in the most proximal airway (corresponding to generations 3-5) was 8.1 ± 0.3 mm (N = 8) and 6.0 ± 0.2 mm in the most distal airway (generations 8-11). Subsurface structures such as cartilage plates are also detectable illustrating the potential of *a*OCT for airway wall measurements such as wall thickness [23,25], though this function was not evaluated in the present study.

**Figure 4:**
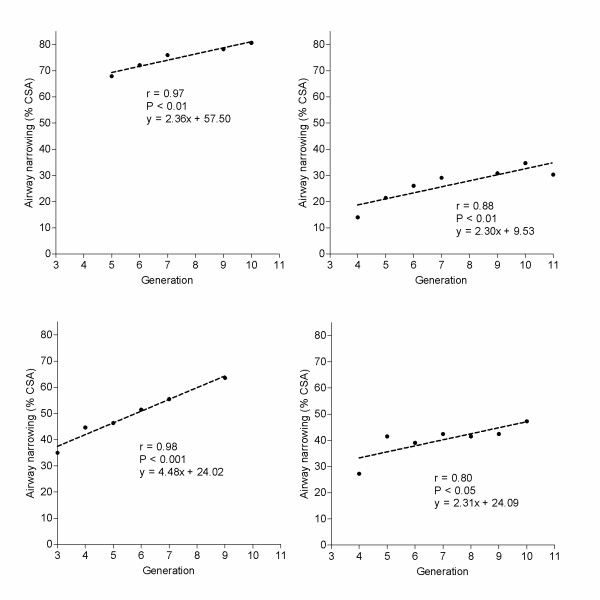
**Relationship between generation and airway narrowing to carbachol administered to the adventitial airway surface**. Plots are from four different airway preparations. Airway narrowing was quantified by the percentage decrease in luminal cross sectional area (% CSA). Airway narrowing was increased at more distal locations indicated by positive correlations between narrowing and airway generation in each preparation. Linear equations of best fit are indicated for each airway. (*Pearson's correlation analysis*)

Scans were acquired along the entire length of the airway preparation before and after carbachol and changes in cross sectional area were measured until the narrowing to carbachol had reached a minimum cross sectional area. Airway narrowing to carbachol administered to either adventitial or luminal surfaces produced a heterogeneous pattern of airway narrowing, such that narrowing was more pronounced at distal locations. Furthermore, there was a close correlation between airway generation and narrowing (Analysis 1) by the adventitial route in all preparations investigated (Figure 4, N = 4). In comparison, when carbachol was administered to the luminal surface in a different set of four airways, heterogeneity in airway narrowing between different generations was less pronounced. Only one out of four airways investigated showed a significant relationship between airway narrowing and generation: r = 0.91, 4 data points, NS; r = 0.34, 5 data points, NS; r = 0.52, 6 data points, NS; r = 0.82, 6 data points, P < 0.05.

**Figure 5:**
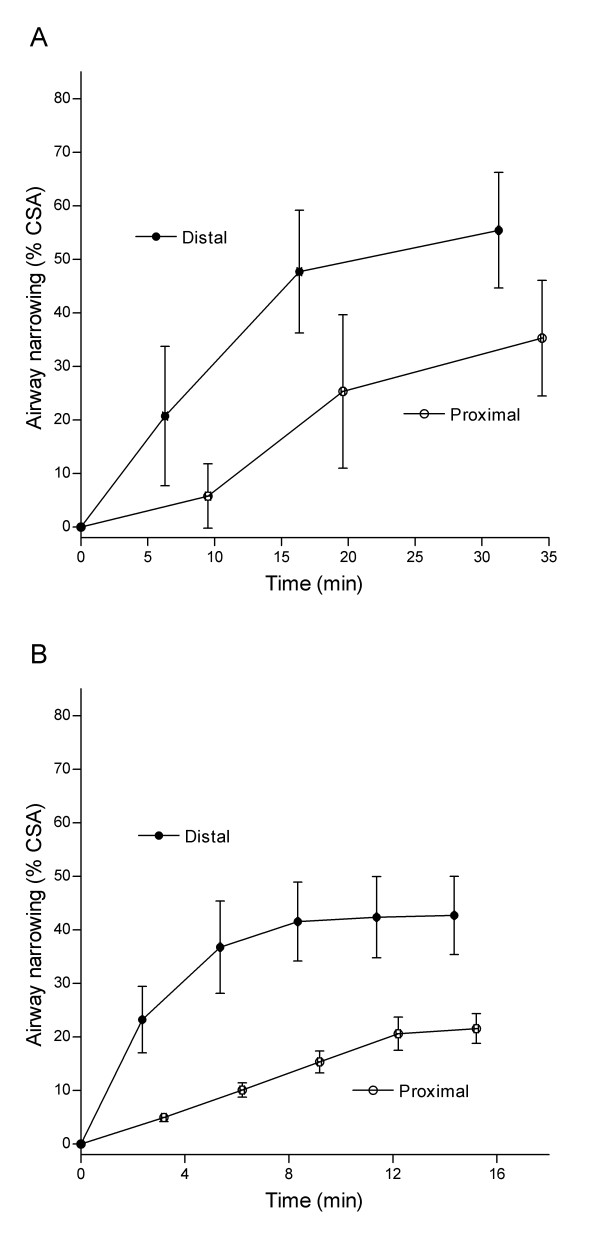
**Airway narrowing measured in the most distal and proximal airways within the bronchial airway preparation**. Airway narrowing was induced by carbachol administered to either (A) adventitial (N = 4) or (B) luminal (N = 4) airway surface. Airway narrowing was quantified from the percentage decrease in luminal cross sectional area (% CSA). Airway narrowing recordings were somewhat later in the proximal airway since *a*OCT scans were initiated from the distal airway (i.e., proximal recordings occurred ~1 min and 3 min later for luminal and adventitial protocols, respectively). Airway narrowing was greater in distal airways irrespective of whether the drug was applied to the adventitial (P < 0.05) or luminal (P < 0.001) surface. (*Two-way ANOVA*).

As a result of the progressive increase in narrowing with generation there were substantial differences in airway narrowing to carbachol in the most distal airway compared to the most proximal, irrespective of the route of drug administration (Figure 5A and 5B). Accordingly, when carbachol was administered adventitially, the maximum reduction in lumen area was 55.4 ± 10.8% in the distal airway, and 36.0 ± 11.5% in the proximal airway (P < 0.05, N = 4). Added to the luminal surface, the maximum reduction in lumen area was 43.2 ± 7.5% and 22.1 ± 3.0% in the distal and proximal airway, respectively (P < 0.01, N = 4). There were no differences in maximum narrowing responses between adventitial or luminal drug administration.

The rate of airway narrowing also varied with airway generation and was more rapid in distal than proximal airways as indicated by negative correlations in the time to 50% response for adventitial drug administration in all four preparations investigated (Table 1). By that route, the time to 50% maximum response was 9.2 ± 2.3 min at the furthermost distal airway, which was significantly less than 19.0 ± 2.4 min recorded at the furthermost proximal airway (P < 0.01, N = 4). Although there was a trend for faster narrowing to luminal carbachol in distal airways, rates (i.e., 2.8 ± 0.7 min and 6.8 ± 0.8 min for distal and proximal airways, respectively) and correlations were not statistically significant. As reported previously [24], the rate of narrowing was greater when carbachol was added to the luminal surface than to the adventitial surface (P < 0.01).

**Table 1:** Correlation coefficients for airway generation against time to 50% response

Adventitia	Lumen
-0.89, P < 0.05, N = 5	-0.87, NS, N = 4

-0.76, P < 0.05, N = 7	-0.54, NS, N = 5

-0.86, P < 0.05, N = 6	-0.71, NS, N = 6

-0.97, P < 0.001, N = 7	-0.56, NS, N = 6

Data are from eight different airway preparations that were constricted to carbachol administered either to the adventitial or luminal airway surface. Time to 50% response of airway narrowing was used as an index of the rate of airway narrowing and was plotted against airway generation. When carbachol was administered to the adventitial surface time to 50% response was negatively correlated with airway generation indicating a faster rate of narrowing at distal locations. In comparison, when carbachol was added to the luminal surface correlations were not significant. N = number of data points available for each individual airway. (*Pearson's correlation analysis*)

**Figure 6:**
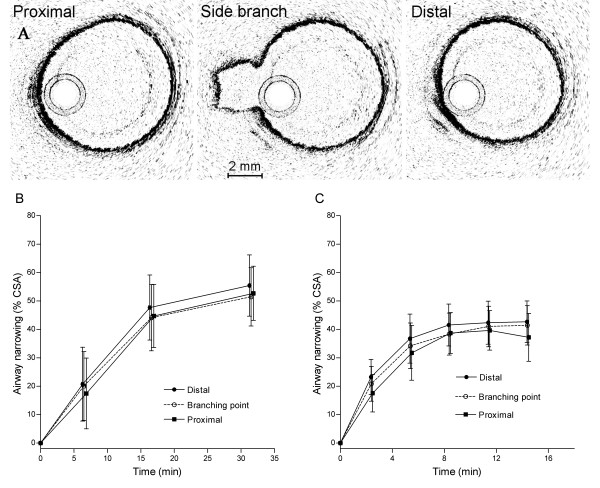
**Airway narrowing within the parent bronchus at branching sites**. (A) Cross sectional images of a relaxed parent bronchus at a branching site and immediately proximal and distal to the branching site. The parent bronchus opens out to the daughter airway at the centre of the branching site. Airway narrowing to adventitial (B, N = 4) and luminal carbachol (C, N = 4) was compared at the three locations identified in (A). There was no difference in narrowing of the parent bronchus between branching or non branching sites irrespective of whether carbachol was administered to the adventitial or luminal surface. (*Two-way ANOVA*).

In addition to the measurements of airway narrowing at different generations within the parent bronchus (as in Analysis 1 above), airway narrowing was also measured at regions where branching occurred. We assessed whether structural variations at regions of branching modified airway narrowing within the parent bronchus itself (Analysis 2). Airway narrowing was measured in the parent bronchus either side of the branching site, and at the mid point where the parent bronchus opened into the side branch (Figure 6A). For measurements at branching sites, the process of manually tracing the airway lumen involved some interpolation due to the opening of the side branch mouth. No differences in airway narrowing were observed at branching versus non branching sites either when carbachol was added to the adventitial (Figure 6B) or to the luminal surface (Figure 6C).

**Figure 7:**
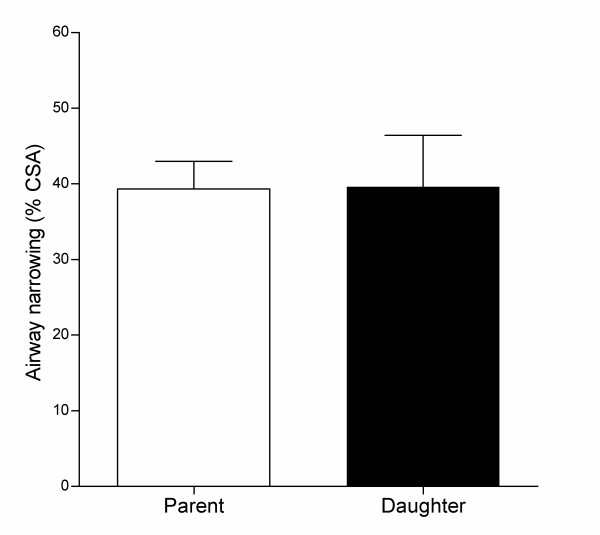
**Comparison of airway narrowing in parent and daughter bronchi**. Airway narrowing was measured in response to luminally applied carbachol in the mouth of a daughter side branch and in the parent bronchus at the branching site. A total of 21 parent-daughter branching sites were measured from four different airway preparations. There was no difference in airway narrowing between parent and daughter bronchi. *(Paired t-test)*.

A further analysis was performed to compare airway narrowing in the mouth or opening of daughter bronchi and the parent bronchus (Analysis 3). Daughter bronchi (i.e., side branches) were readily visible on 3D reconstructions of the airway preparation (Figure 1). Airway narrowing to carbachol was measured in a total of 21 parent and daughter bronchi from four airway preparations. The magnitude of airway narrowing measured in daughter bronchi was the same as that measured in the parent bronchus (Figure 7).

**Figure 8:**
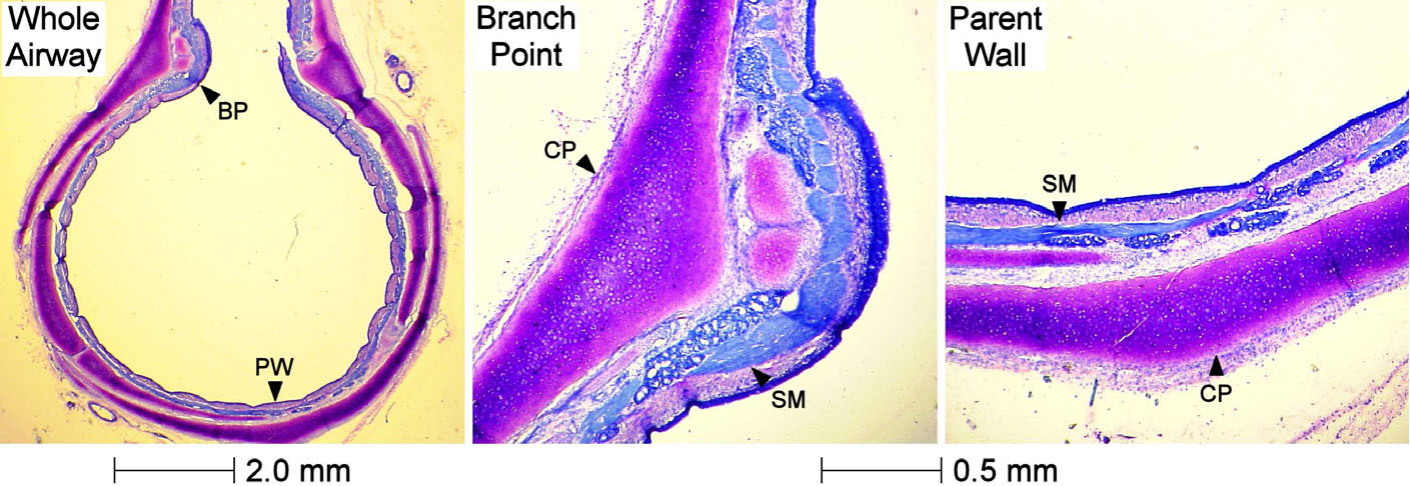
**A histological cross section of a parent bronchus at a point of branching**. The entire cross section of the bronchus is shown (Left panel, Whole Airway) indicating the point at which the parent bronchus opens out into the daughter. The parent bronchial wall (PW) and the wall at the shoulder of a branching point (BP) are indicated by arrows. Middle and right panels are magnified images of the branching point and of the parent bronchial wall respectively. Substantial thickening of the cartilage plate (CP) at the branching point is apparent, which is accompanied by an increase in smooth muscle (SM) mass and a more oblique orientation of muscle cells.

Finally, in order to identify possible structural properties that may influence airway narrowing at regions of branching, histological cross sections of parent bronchi were obtained at branching points (see example in Figure 8.). The figure indicates considerable thickening of the cartilage at the junction between the parent and daughter bronchus. Cartilage thickening was accompanied by an apparent increase in ASM mass. To investigate the consistency of this observation, we conducted a semi-quantitative analysis in a random sample of seven side branches from seven airways. In six of these bronchi both thicker cartilage and increased ASM at the side branch were scored, compared to the adjacent parent bronchus. One bronchus showed little change in either cartilage or ASM. In all seven airways, ASM was aligned obliquely at the side branch in comparison to parent airway wall regions.

## Discussion

*a*OCT provides a novel and advanced means of imaging the airway lumen with sufficiently short acquisition times to enable the spatial and temporal response to bronchoconstrictor stimuli to be recorded. The scanning pullback protocol of *a*OCT enables a large region of the airway to be investigated essentially in 3D, unlike other techniques such as X-ray radiography and videoendoscopy that produce 2D images at single locations in the airway. In applying *a*OCT to the study of airway physiology we have demonstrated relationships between airway stimulation and the dynamic response at defined, identifiable and contiguous regions of the bronchial tree across a wide range of generations ~3 to 11. Importantly, we investigated regional airway narrowing in more depth than has previously been undertaken, including narrowing at side branches, which even in the face of known structural differences at these regions [15-18], has not been reported elsewhere. The study shows a progressive increase in airway narrowing from the proximal to distal airway, a relationship that was influenced by the route of drug administration but not by structural variations accompanying branching sites.

Despite many studies endeavoring to characterize the distribution of airway narrowing responses throughout the lung, the regional distribution of such responses has still not been clearly defined. Whilst some studies report the greatest narrowing in proximal airways [10,11], others report it in more distal generations [7] or somewhere in between [14]. It is likely that most of these inconsistencies can be attributed to methodological complexities or limitations. For example narrowing responses in central versus peripheral airways could be influenced by variations in the deposition of bronchoconstrictor drugs [11,12] and by uncertainty in the anatomical relationships between large and small diameter airways [3-7]. In light of these shortcomings, an intention of the present study was to assess anatomical variations in the intrinsic narrowing capacity of airways using an experimental design that ensured different airway generations were assessed under identical mechanical and physiological conditions. A range of identifiable airway generations were imaged within a short time window, which allowed narrowing responses in those generations to be recorded almost concurrently, thereby establishing both a spatial and temporal relationship of response. The isolated airway preparation was free of parenchymal attachments and had a standardized transmural pressure (pre and afterload) and volume history so that differences could be attributed to the intrinsic properties of the airway alone. Our findings suggest that peripherally, airways become intrinsically more responsive to cholinergic stimulation, broadly confirming earlier reports in pig bronchi *in vitro *in which airway narrowing was measured endoscopically in separate large and small diameter airways [7]. The present study extends these findings by documenting narrowing over a wide range of identified interconnecting airway generations. We show that static and dynamic narrowing responses to carbachol administered to the airway adventitia correlate specifically to contiguous and defined airway generations, i.e., airway narrowing within the bronchial tree exhibits serial heterogeneity.

In separate experiments we also examined airway narrowing responses to carbachol administered to the airway lumen which replicates the physiological condition in provocation testing. Although heterogeneity was also shown with luminal carbachol, it was less pronounced than exposure via the adventitia, particularly generation by generation, suggesting that the intrinsic response of the airway wall was suppressed or regulated by some property of the epithelium or mucosa. Note that when drugs are administered via the adventitia, there is unhindered access to the ASM. In contrast, when administered through the lumen, tight junctions in the epithelium are impermeable to contractile drugs such as carbachol [24] and, as a result, the epithelial barrier will strongly regulate airway narrowing. It was for this reason that we used a higher concentration of carbachol in the lumen than adventitia in order to obtain a comparable level of bronchoconstriction. The precise mechanisms whereby the epithelium or mucosa might regulate the expression of the intrinsic heterogeneity to luminal activation are not clear. However, we suggest that the permeability of the epithelium is potentially important in this respect by regulating airway constrictor responses and the distribution of airway narrowing throughout the lung.

In this study airway narrowing in different airway generations was measured in response to a single dose of carbachol. Under these conditions, differences in airway narrowing to bronchoconstrictor stimuli could reflect differences in sensitivity and/or reactivity, the two broad determinants of airway responsiveness. Separation of these variables requires the construction of complete dose response curves, which is impracticable even using *a*OCT. Intrinsic differences in ASM sensitivity to carbachol would principally determine airway sensitivity, whilst differences in ASM mass or stress and the load against which ASM shortens could regulate the degree and rate of narrowing [26]. In the present study, we favor the latter explanation of a shift in airway reactivity as the source of heterogeneity because the sensitivity and stress of isolated ASM to cholinergic agonists is very similar in bronchi and bronchioles in this species [27,28]. Furthermore, a previous study demonstrated greater maximum narrowing to acetylcholine in small diameter pig airways than in large [7]. Whilst future studies are required to identify the precise mechanism responsible for variations in the degree and rate of narrowing demonstrated in this study, there may be differences in the mechanical properties/behavior of the airway wall between generations. The airway wall is a multilayered structure comprising mucosa, ASM and cartilage with fibroelastic connections between the layers and each is stressed in response to bronchoconstrictor stimulation [7,29,30]. Airway narrowing will be subject to their respective elastic moduli and these may vary at different locations within the bronchial tree and produce the serial heterogeneity observed in the present study.

Based on the relationship between airway narrowing and generation observed here, we would predict that airway closure would only occur, if at all, in the most peripheral airway generations. Airway closure *in vivo *will of course have significant physiological consequences, including lung hyperinflation, an increased work of breathing and an impaired gas exchange, all of which occur in obstructive respiratory disease (asthma). While airway closure was not observed in this study, it is important to appreciate that flow may well be reduced to physiologically unsustainable levels prior to absolute airway closure, that is 'functional closure'[7]. Moreover any relationship between airway narrowing and generation could change considerably in disease as a result of inflammation and airway wall remodeling [31], manifested by a change in the slope of the relationship or perhaps a shift in the point of airway closure to more proximal lung generations [32]. However there are also several other factors that will influence the relationship between airway narrowing and generation, most notably ASM activation, which in the present study was approximately half that possible with exogenous carbachol. The relationship between airway narrowing and generation could further vary with other modes of stimulation to those investigated here, for example, airway narrowing induced by parasympathetic nerves, which will depend on the distribution and density of nerve endings. In an early study by Cabezas and colleagues [33], bronchoconstriction to vagal stimulation showed greater responses to stimulation in smaller airways than large, possibly for the above reason, or as favored by the present study, due to other intrinsic differences between small and large airways. Finally, differences in branching pattern and airway wall composition between species will likely impact on the distribution of airway narrowing responses to ASM activation.

Previous attempts have been made to identify serial heterogeneity within the bronchial tree. However, to our knowledge no study has determined whether more localized heterogeneity exists at side branches. Indeed, branching points might have been considered an obstacle to be avoided in past studies. By constructing both 2D and 3D images, we provide the first information on narrowing at the entrance of the daughter airway and whether the presence of a side branch affects narrowing in the parent trunk. The results of the analysis have shown that branching has no major effect on airway narrowing; responses were uniform both within the trunk of the parent airway and in the entrance, or mouth, of an emerging side branch. Given the structural complexity of branching, which requires a transition in ASM orientation and cartilage, each of which could potentially affect airway narrowing, these findings are somewhat surprising. In the monopodial airway of the pig, and other species, daughter airways emerge from the parent trunk and the predominant circumferential orientation of ASM in the parent re-orientates around the daughter [17,18], which might reduce circumferential active stress in both parent and daughter. Saddle-like plates of cartilage support the structural division [15,16] and the additional mechanical load might be expected to restrict airway narrowing [34,35].

A number of explanations can be offered as to why airway narrowing is maintained at regions of branching. One may be that the structural complexity at the dividing point is too localized to exert an overall effect on airway function, e.g., active ASM stress from neighboring parts of the airway prevails over any lack of stress at the bifurcation. Importantly, a strength of our approach was that we preserved the 3D structural integrity of the airway wall so that the physiological behavior of the entire airway wall could be determined. Secondly, a semi-quantitative analysis of airways used in the present study indicated an abundance of ASM as well as cartilage in the immediate point of branching, which could explain why airway narrowing responses were maintained at these regions. We speculate that a greater ASM mass at a bifurcation would develop more force than elsewhere in the airway tree, compensating for any change in ASM orientation that necessarily accompanies transition from parent to daughter airway, or any additional load associated with the cartilage in the dividing airway wall [34,35].

In summary, *a*OCT images provide a comprehensive view of contiguous regions of the bronchial tree enabling both steady state and dynamic responses of airway narrowing to be followed in identifiable and defined portions of the tree. Although the bronchial tree exhibits an intrinsic and progressive increase in the rate and amount of narrowing to carbachol as it extends peripherally, the expression of this physiological heterogeneity is partly offset when drugs are administered via the lumen and epithelium. *a*OCT-derived 3D images also provided the first measurements of airway narrowing in and around side branches. Our findings are that airway narrowing is not affected by structural complexities produced by branching, as indicated by similar narrowing between parent and daughter airways.

## List of Abbreviations

*a*OCT: anatomical optical coherence tomography; ASM: airway smooth muscle; FEV_1_: forced expiratory volume in 1 sec; OD: outer diameter.

## Competing interests

JJA, PRE, DRH and DDS are listed as inventors on a provisional patent application associated with clinical applications of anatomical optical coherence tomography. RAM is listed as a potential beneficiary of this same provisional patent application. The other authors have no competing interests regarding this study.

## Authors' contributions

PBN was responsible for experimental design, data collection/analysis, and also preparation of the manuscript. RAM was responsible for the development of the quantification software, and assisted in data collection and manuscript preparation. ARW assisted in data collection, manuscript preparation and contributed to experimental design. SB and JJA assisted in data collection. PKM, PRE, DRH, and DDS provided intellectual input and assisted in manuscript preparation. HWM was responsible for experimental design and manuscript preparation. All authors read and approved the final manuscript.

## References

[B1] King GG Carroll JD Muller NL Whittall KP Gao M Nakano Y Paré PD Heterogeneity of narrowing in normal and asthmatic airways measured by HRCT Eur Respir J 2004 24 2 211 218 10.1183/09031936.04.00047503 15332387

[B2] Venegas JG Winkler T Musch G Vidal Melo MF Layfield D Tgavalekos N Fischman AJ Callahan RJ Bellani G Harris RS Self-organized patchiness in asthma as a prelude to catastrophic shifts Nature 2005 434 7034 777 782 10.1038/nature03490 15772676

[B3] Amirav I Kramer SS Grunstein MM Hoffman EA Assessment of methacholine-induced airway constriction by ultrafast high-resolution computed tomography J Appl Physiol 1993 75 5 2239 2250 8307884 10.1152/jappl.1993.75.5.2239

[B4] Brown RH Herold CJ Hirshman CA Zerhouni EA Mitzner W In vivo measurements of airway reactivity using high-resolution computed tomography Am Rev Respir Dis 1991 144 1 208 212 2064130 10.1164/ajrccm/144.1.208

[B5] Brown RH Herold CJ Hirshman CA Zerhouni EA Mitzner W Individual airway constrictor response heterogeneity to histamine assessed by high-resolution computed tomography J Appl Physiol 1993 74 6 2615 2620 8365960 10.1152/jappl.1993.74.6.2615

[B6] Dandurand RJ Wang CG Phillips NC Eidelman DH Responsiveness of individual airways to methacholine in adult rat lung explants J Appl Physiol 1993 75 1 364 372 8376287 10.1152/jappl.1993.75.1.364

[B7] Mitchell HW Cvetkovski R Sparrow MP Gray PR McFawn PK Concurrent measurement of smooth muscle shortening, lumen narrowing and flow to acetylcholine in large and small porcine bronchi Eur Respir J 1998 12 5 1053 1061 10.1183/09031936.98.12051053 9863996

[B8] Murphy TM Roy L Phillips IJ Mitchell RW Kelly EA Munoz NM Leff AR Effect of maturation on topographic distribution of bronchoconstrictor responses in large diameter airways of young swine Am Rev Respir Dis 1991 143 1 126 131 1670916 10.1164/ajrccm/143.1.126

[B9] Nagase T Moretto A Ludwig MS Airway and tissue behavior during induced constriction in rats: intravenous vs. aerosol administration J Appl Physiol 1994 76 2 830 838 8175596 10.1152/jappl.1994.76.2.830

[B10] Okazawa M Bai TR Wiggs BR Paré PD Airway smooth muscle shortening in excised canine lung lobes J Appl Physiol 1993 74 4 1613 1621 8514675 10.1152/jappl.1993.74.4.1613

[B11] Okazawa M Vedal S Verburgt L Lambert RK Paré PD Determinants of airway smooth muscle shortening in excised canine lobes J Appl Physiol 1995 78 2 608 614 7759430 10.1152/jappl.1995.78.2.608

[B12] Opazo Saez A Du T Wang NS Martin JG Methacholine-induced bronchoconstriction and airway smooth muscle in the guinea pig J Appl Physiol 1996 80 2 437 444 8929581 10.1152/jappl.1996.80.2.437

[B13] Shioya T Solway J Munoz NM Mack M Leff AR Distribution of airway contractile responses within the major diameter bronchi during exogenous bronchoconstriction Am Rev Respir Dis 1987 135 5 1105 1111 3555187 10.1164/arrd.1987.135.5.1105

[B14] Bai Y Zhang M Sanderson MJ Contractility and Ca2+ signaling of smooth muscle cells in different generations of mouse airways Am J Respir Cell Mol Biol 2007 36 1 122 130 10.1165/rcmb.2006-0036OC 16931808 PMC1899303

[B15] von Hayek H The human lung 1960 New York: Hafner Pub. Co

[B16] Reid L Visceral cartilage J Anat 1976 122 2 349 355 794047 PMC1231907

[B17] Smiley-Jewell SM Tran MU Weir AJ Johnson ZA Van Winkle LS Plopper CG Three-dimensional mapping of smooth muscle in the distal conducting airways of mouse, rabbit, and monkey J Appl Physiol 2002 93 4 1506 1514 12235053 10.1152/japplphysiol.01109.2001

[B18] Sparrow MP Lamb JP Ontogeny of airway smooth muscle: structure, innervation, myogenesis and function in the fetal lung Respir Physiol Neurobiol 2003 137 2-3 361 372 10.1016/S1569-9048(03)00159-9 14516738

[B19] Noble PB Sharma A McFawn PK Mitchell HW Airway narrowing in porcine bronchi with and without lung parenchyma Eur Respir J 2005 26 5 804 811 10.1183/09031936.05.00065405 16264040

[B20] Armstrong JJ Leigh MS Sampson DD Walsh JH Hillman DR Eastwood PR Quantitative upper airway imaging with anatomic optical coherence tomography Am J Respir Crit Care Med 2006 173 2 226 233 10.1164/rccm.200507-1148OC 16239620

[B21] Walsh JH Leigh MS Paduch A Maddison KJ Philippe DL Armstrong JJ Sampson DD Hillman DR Eastwood PR Evaluation of pharyngeal shape and size using anatomical optical coherence tomography in individuals with and without obstructive sleep apnoea J Sleep Res 2008 17 2 230 238 10.1111/j.1365-2869.2008.00647.x 18422508

[B22] McLaughlin RA Williamson JP Phillips MJ Armstrong JJ Becker S Hillman DR Eastwood PR Sampson DD Applying anatomical optical coherence tomography to quantitative 3D imaging of the lower airway Opt Express 2008 16 22 17521 17529 10.1364/OE.16.017521 18958032

[B23] Noble PB West AR McLaughlin RA Armstrong JJ Becker S McFawn PK Williamson JP Eastwood PR Hillman DR Sampson DD Mitchell HW Airway narrowing assessed by anatomical optical coherence tomography in vitro: dynamic airway wall morphology and function J Appl Physiol 2009 19910337 10.1152/japplphysiol.00511.2009

[B24] Sparrow MP Mitchell HW Modulation by the epithelium of the extent of bronchial narrowing produced by substances perfused through the lumen Br J Pharmacol 1991 103 1 1160 1164 1878753 10.1111/j.1476-5381.1991.tb12317.x PMC1908092

[B25] Coxson HO Quiney B Sin DD Xing L McWilliams AM Mayo JR Lam S Airway wall thickness assessed using computed tomography and optical coherence tomography Am J Respir Crit Care Med 2008 177 11 1201 1206 10.1164/rccm.200712-1776OC 18310475 PMC2408438

[B26] Macklem PT Mechanical factors determining maximum bronchoconstriction Eur Respir J Suppl 1989 6 516s 519s 2803407

[B27] Gray PR Mitchell HW Effect of diameter on force generation and responsiveness of bronchial segments and rings Eur Respir J 1996 9 3 500 505 10.1183/09031936.96.09030500 8730010

[B28] Sparrow MP Mitchell HW Contraction of smooth muscle of pig airway tissues from before birth to maturity J Appl Physiol 1990 68 2 468 477 2318758 10.1152/jappl.1990.68.2.468

[B29] Olsen CR Stevens AE Pride NB Staub NC Structural basis for decreased compressibility of constricted tracheae and bronchi J Appl Physiol 1967 23 1 35 39 6028161 10.1152/jappl.1967.23.1.35

[B30] Mitchell HW Gray PR Uncoupling in the wall of the cartilaginous bronchus of the pig produced by smooth muscle contraction Pulm Pharmacol 1996 9 1 29 34 10.1006/pulp.1996.0003 8843507

[B31] Carroll N Elliot J Morton A James A The structure of large and small airways in nonfatal and fatal asthma Am Rev Respir Dis 1993 147 2 405 410 8430966 10.1164/ajrccm/147.2.405

[B32] Brown RH Pearse DB Pyrgos G Liu MC Togias A Permutt S The structural basis of airways hyperresponsiveness in asthma J Appl Physiol 2006 101 1 30 39 10.1152/japplphysiol.01190.2005 16469934

[B33] Cabezas GA Graf PD Nadel JA Sympathetic versus parasympathetic nervous regulation of airways in dogs J Appl Physiol 1971 31 5 651 655 4399001 10.1152/jappl.1971.31.5.651

[B34] Jiang H Stephens NL Contractile properties of bronchial smooth muscle with and without cartilage J Appl Physiol 1990 69 1 120 126 2394641 10.1152/jappl.1990.69.1.120

[B35] Noble PB Turner DJ Mitchell HW Relationship of airway narrowing, compliance, and cartilage in isolated bronchial segments J Appl Physiol 2002 92 3 1119 1124 11842048 10.1152/japplphysiol.00662.2001

